# Development Projects to Improve Maternal and Child Health: Assessing the Impact

**DOI:** 10.1371/journal.pmed.0030192

**Published:** 2006-04-18

**Authors:** Yemane Berhane

## Abstract

Berhane discusses a new study in *PLoS Medicine* that evaluated the effect of improved water access in rural Ethiopia upon birth rate and child malnutrition.

Demographic and social conditions in Ethiopia pose serious challenges to human survival. These challenges are particularly relevant to women, who often have the prime responsibility for the welfare of children and the family at large. Women's status in Ethiopia, as in other developing countries, is low, and their daily workload is often enormous [ 1]. Women have the dual responsibility of both reproducing and ensuring the survival of their children. Leisure time for women is declining due to increasing economic difficulties; even the period of confinement following childbirth has declined [ 1].

There have been many initiatives to improve the life of people living in rural Ethiopia over the last few decades, but many of these initiatives were not systematically evaluated. Thus, the short-term and long-term impacts of development interventions in Ethiopia remain largely unknown or unreported. In a new study in *PLoS Medicine*, Mhairi Gibson and Ruth Mace attempted to evaluate the effect of improved water access in rural Ethiopia—a development initiative that reduces women's workloads since they no longer have to collect water from distant sites—on birthrate and child malnutrition [ 2]. As such, this study is a very important step forward in making developmental interventions evidenced based and contextual.

## The Study Findings

Gibson and Mace collected retrospective demographic data over a four-year period in eight rural villages in Arsi zone, Ethiopia. These included villages with and without the water development scheme. Villages with and without tapped water were comparable with respect to size, altitude, ethnicity, and religion. Using logistic hazards models and general linear modeling techniques, the authors found an association between the development initiative and a decline in infant mortality. However, the initiative was also associated with an increase in birthrate. And the initiative was not associated with any improvement in maternal or child health or in nutritional status. In fact, after controlling for any effects of age, sex, birth order, socioeconomic status, and family size, children living in villages with water access were at a significantly greater risk of being malnourished and stunted.

The impacts of development interventions in Ethiopia remain largely unknown or unreported.

In summary, the study showed that developmental interventions may have deleterious effects on overall societal well-being in the long run if they are not implemented and monitored carefully. In this case, the increased birthrate could potentially exacerbate the population pressure on the already scarce farming land.

## Limitations of the Study

Although the study has many strong methodological aspects, including the use of a local event calendar to obtain reliable data and advanced statistical techniques to analyze the data, there are also a number of limitations. First, the retrospective nature of the data collection in a rural and largely illiterate population is a major limitation. Retrospective birth history data collection is problematic—many women fail to report unsuccessful pregnancies and early infant deaths [ 3]. Also, a full account of the development intervention is not given; for instance, how often did women fetch water in the intervention and nonintervention villages? what proportion of their time was saved by the intervention? and where did women fetch water during the rainy season? All these factors are important in relation to the intervention.

In addition, the authors did not compare study participants at an individual level in the intervention and nonintervention villages for various demographic and social factors. In rural populations, childbirth is heavily influenced by interrelated social and economic factors—which need to be taken into account [ 4]. Anthropometric assessments were done only once during the study period, which is not sufficient because of marked seasonal and annual variations. These variations are particularly likely to occur in drought-prone areas, such as the study area. There are marked seasonal variations in Ethiopia in terms of food availability and safety of drinking water [ 5]. The nutritional status of Ethiopian children is very poor and is affected by many factors other than water access, including food production, prevalence of infectious diseases, and frequent drought and famine [ 6, 7].

## Policy Implications

The study offers a very strong message, particularly to policymakers, about the need for an integrated approach to development interventions. Development projects that focus on a solitary technological intervention cannot improve the health status of women and society at large. For instance, a study in another rural population of Ethiopia showed that improved supply of safe water without improved sanitation had little effect on the health status of children [ 8]. Gibson and Mace's study also provides good evidence for health-service providers to be vigilant in local interventions and show interest in proper evaluation of the health and demographic consequences of developmental interventions.

There are three important aspects in the assessment of development interventions that need further work. First, evaluation of developmental interventions needs to be based on prospective collection of data in order to control for extraneous factors that influence the effect of the intervention. Second, further study is needed to better understand the mechanism by which time saved due to improved access to a water source is translated into increased childbirth rate. Finally, since anthropometric parameters show seasonal and annual variations, especially in drought-prone areas, nutritional assessments need to be done regularly and over a long period of time.

## References

[pmed-0030192-b1] Berhane Y, Gossaye Y, Emmelin M, Hogberg H (2001). Women's health in rural setting in societal transition in Ethiopia. Soc Sci Med.

[pmed-0030192-b2] Gibson MA, Mace R (2006). An energy-saving development initiative increases birth rate and childhood malnutrition in rural Ethiopia. PLoS Med.

[pmed-0030192-b3] Central Statistical Authority (2000). Central Statistical Authority bulletin 228: Analytical report on the 1998/99 sample survey and vital events registration.

[pmed-0030192-b4] Boserup E (1985). Economic and demographic interrelationships in sub-Saharan Africa. Popul Dev Rev.

[pmed-0030192-b5] Chamber R, Longhurst R, Bradley D, Feachem R (1982). Seasonal dimension to rural poverty: Analysis and implications. J Trop Med Hyg.

[pmed-0030192-b6] Haidar J, Tsegaye T (1999). Nutrition situation in Ethiopia. S Afr Med J.

[pmed-0030192-b7] Getahun Z, Urga K, Genebo T, Nigatu A (2001). Review of the status of malnutrition and trends in Ethiopia. Ethiop J Health Dev.

[pmed-0030192-b8] Asnake M, Larson C, Teka GE (1992). Water handling practices and their association with childhood diarrhea. Ethiop J Health Dev.

